# Nonequilibrium Brownian Motion beyond the Effective Temperature

**DOI:** 10.1371/journal.pone.0093720

**Published:** 2014-04-08

**Authors:** Andrea Gnoli, Andrea Puglisi, Alessandro Sarracino, Angelo Vulpiani

**Affiliations:** 1 Istituto dei Sistemi Complessi - Consiglio Nazionale delle Ricerche, Rome, Italy; 2 Dipartimento di Fisica, Università “Sapienza”, Rome, Italy; 3 Laboratoire de Physique Théorique de la Matière Condensée - Centre National de la Recherche Scientifique Unité mixte de recherche 7600, Université Paris 6, Paris, France; Centre de Physique Théorique, France

## Abstract

The condition of thermal equilibrium simplifies the theoretical treatment of fluctuations as found in the celebrated Einstein’s relation between mobility and diffusivity for Brownian motion. Several recent theories relax the hypothesis of thermal equilibrium resulting in at least two main scenarios. With well separated timescales, as in aging glassy systems, equilibrium Fluctuation-Dissipation Theorem applies at each scale with its own “effective” temperature. With mixed timescales, as for example in active or granular fluids or in turbulence, temperature is no more well-defined, the dynamical nature of fluctuations fully emerges and a Generalized Fluctuation-Dissipation Theorem (GFDT) applies. Here, we study experimentally the mixed timescale regime by studying fluctuations and linear response in the Brownian motion of a rotating intruder immersed in a vibro-fluidized granular medium. Increasing the packing fraction, the system is moved from a dilute single-timescale regime toward a denser multiple-timescale stage. Einstein’s relation holds in the former and is violated in the latter. The violation cannot be explained in terms of effective temperatures, while the GFDT is able to impute it to the emergence of a strong coupling between the intruder and the surrounding fluid. Direct experimental measurements confirm the development of spatial correlations in the system when the density is increased.

## Introduction

Several fundamental results of statistical mechanics are obtained under the crucial assumption of thermal equilibrium. A celebrated example of the power of the equilibrium hypothesis is given by the theoretical treatment of Brownian motion developed by Einstein at the beginning of the 20th century [1]. Such a hypothesis imposes symmetry under time-reversal and offers crucial shortcuts in computations. For instance, in calculating the self-diffusion coefficient, thermal equilibrium provides a simple expression for the osmotic pressure of suspended particles. Later, in Langevin’s approach, the simplification comes from the energy equipartition which determines straightforwardly the mean kinetic energy of Brownian particles. The subsequent evolution of the linear response theory has been entirely based upon equilibrium which is the root of the celebrated Fluctuation-Dissipation Theorem (FDT) [2]. This theorem states that whenever an equilibrium system with Hamiltonian *H*, at temperature *T*, is perturbed in such a way that its Hamiltonian changes into 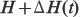, with 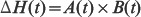 (*B* being a state function of the system, coupled with the external force 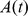), then the mean linear response for the average time evolution of an observable 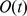 reads
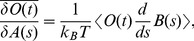
(1)where 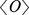 is the average of the unperturbed system, 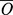 is the perturbed one and 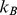 is the Boltzmann’s constant. If the system is invariant for time-translations, in Eq. (1) the times 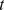 and 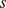 may be replaced by 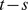 and 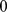, respectively. The FDT is a powerful tool which allows the computation of the effect of small external forces, for instance all transport coefficients, while ignoring such forces. If, for instance, the system is perturbed by an impulse 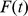 at time 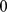, with 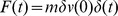 (which in the Hamiltonian appears coupled with 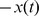) applied to a particle of mass 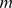, position 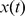 and velocity 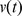, the FDT reads



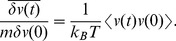
(2)Eq. (2), time-integrated in 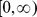, returns Einstein’s relation between diffusivity and mobility.

Recently, it has been shown that even in out-of-equilibrium systems a relation between response and spontaneous fluctuations still exists [3], [4] which takes a more complicated form than the one at equilibrium. An important instance is represented by spin and structural glasses which, cooled below the glass transition temperature, display an extremely slow relaxation called aging [5]. A fundamental observation is that, in some cases, timescales of relevant degrees of freedom are separated into almost perfectly isolated classes, i.e. very fast and very slow evolutions, and an appropriate description of the system can be formulated by introducing the concept of “effective” temperature [6]. For instance, in several models, it has been shown that the FDT, Eq. (1), can be considered as a good approximation, by replacing *T* with an effective time-dependent temperature 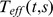 which, for large times, assumes a thermodynamic meaning [7]. Experimental verifications of this scenario have been reported [8], [9]. For driven systems, like fluids under shear, the effective temperature scenario is expected to hold for slow energy flows, namely for slight stirring (which corresponds to the large time limit of glassy models). In particular, this is the case of weakly shaken “glassy” granular media, with density close to jamming [10], [11].

Often in nonequilibrium systems the different timescales are not clearly separated and the picture in terms of effective temperature does not hold. Instances of this entanglement of scales appear in climate and turbulence [3], as well as among the so-called active fluids. They include compounds of actine filaments, swarms of bacteria, bird flocks or fish schools, assemblies of micro-electro-mechanical systems, collective human dynamics (pedestrians, traffic and so on) [12], [13]. The validity of the concept of effective temperature in active matter is under intense debate, with positive [14], [15] and negative [16], [17] answers. Several general approaches to the FDT in nonequilibrium systems have been proposed recently [3], [18], [19]. Some of these stress the relevance of the unperturbed statistical distribution in phase space which, as a rule, includes both non-Gibbsian contributions and dynamical couplings with usually no role in the FDT at equilibrium. Others, connecting the FDT to entropy production and to the so-called *dynamical activity*, give more importance to the statistical distribution in path space and its simmetries under time-reversal [19]. The formulation of the FDT used in this paper [3], called Generalized FDT (GFDT), for the sake of simplicity here expressed in terms of an impulsive force and velocity measurement, reads.
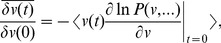
(3)where 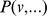 is the unperturbed steady state distribution in the whole phase space, involving all the relevant variables, that is not only the perturbed particle but all the surrounding particles of the fluid. It is clear that at equilibrium, where 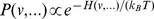, the impulsive form of Einstein’s relation, Eq. (2), is recovered.

## Results and Discussion

**Figure 1:**
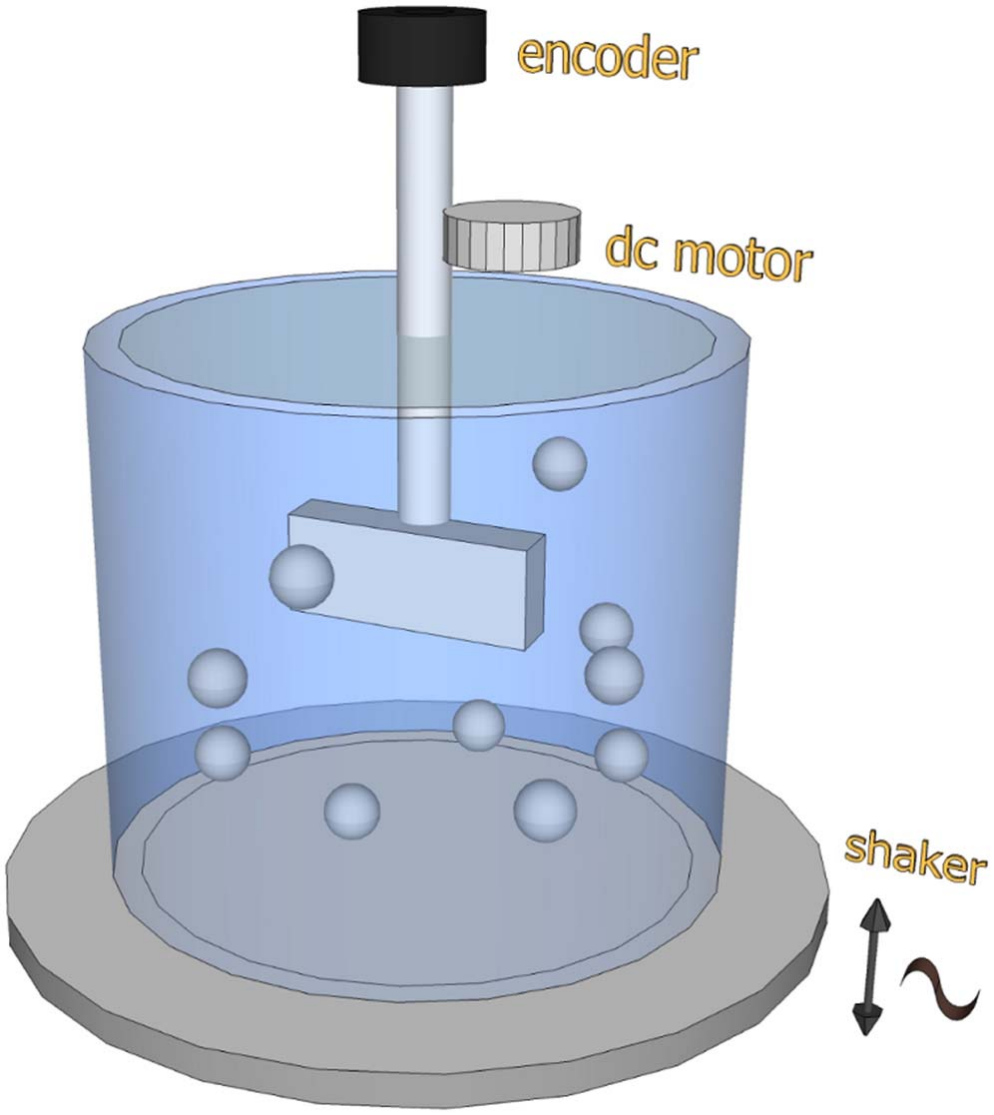
Experimental setup. A sketch of the setup illustrates the essential components. A wheel rotating around a fixed axis is suspended in a cylindrical cell containing steel spheres. The cell is shaken in order to fluidize the material and obtain a granular gas. The wheel performs a Brownian-like dynamics, randomly excited by collisions with the spheres. A small motor is coupled to the wheel axis, in order to apply an external impulsive perturbation. An angular encoder reads the angular velocity of the wheel. Statistical properties of the velocities of the spheres are collected through a fast camera, placed above the system. A detailed description is presented in Methods section.

A paradigmatic case in which Eq. (3) can be tested is that of strongly fluidized granular media [20] for which the overall effect of the energy injection mechanism and the presence of energy exchanges on different space- or time-scales can induce complex behaviors. In such systems, interactions among particles are dissipative due to the energy loss during the collisions and an external source is necessary in order to sustain a fluid stationary state. A strict analogy with simple Brownian motion was shown in a previous work analysing the rotational motion of a torsion oscillator immersed in a dense granular fluid [21]. By measuring noise and susceptibility in the system, the authors found that an effective description can be obtained within the equilibrium formalism and showed that the shaken granular medium acts as a “thermal” bath satisfying the FDT. Here, we consider a new experiment, described in Figure 1 (see section Methods for further details), where a rotating wheel performs granular Brownian motion immersed in a shaken granular media [22] and is weakly perturbed by the impulsive action of a small motor. The motor is switched on for a very short lapse of time, and exerts – at an arbitrary time set to 0 – a variation of the wheel’s angular velocity 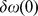. We explore the range of low and medium densities (up to a maximum of 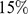 of packing fraction) in order to assess multiscale regimes not considered previously [21].

### Linear Response

**Figure 2:**
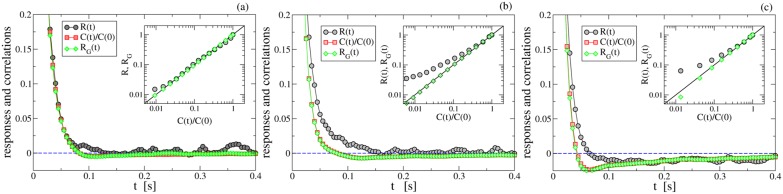
Response and autocorrelation. Response function 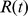 (black circles), rescaled velocity autocorrelation 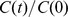 (red squares), and GFDT response with the factorization assumption, Eq. (6), 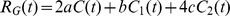 (green diamonds) for 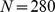 (a), 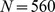 (b) and 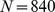 (c), that is packing fractions 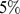, 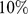 and 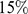, respectively. In the inset the parametric plot 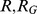 *vs* 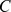, in the region where 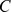 is positive and monotonously decreasing, is plotted in log-log scale. In the densest cases, 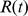 and 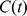 behave very differently and Einstein’s relation is significantly violated.

The measurements of interest in our experiment are the response of the angular velocity 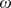 of the wheel to the perturbation, 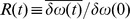, and the time-correlations of the unperturbed signal 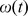, *in primis* the classical auto-correlation 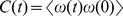. In Fig. 2, for different values of the gas density, we show the results for 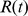 superimposed to 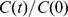. In the dilute limit, panel (a), correlations and response functions are very close, so that 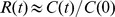 with slight departures which we ascribe to the large noise of the response signal. This observation, even more compelling in the inset of Fig. 2a showing a parametric plot 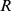 *vs* 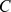, is equivalent to verifying Einstein’s relation, Eq. (2). Note that, normalizing the response function, the measurement of a proportionality factor 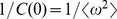 is inevitable even if equipartition is not satisfied (indeed, 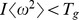 because of inelastic collisions, where 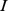 is the momentum of inertia of the wheel and 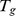 is the granular kinetic temperature). The fact that a Brownian particle suspended in a dilute granular fluid behaves as if it were at equilibrium has been observed before [23]: the separation of scales guaranteed by diluteness allows the granular gas to be considered almost independent upon the dynamics of the wheel; such a decoupling implies that each inelastic collision of the wheel with a gas particle may be understood as an elastic collision with different effective masses.

For higher values of the gas density, panels (b) and (c) of Fig. 2, the scenario changes considerably. Here, the dynamics of the tracer and of the gas have to be considered coupled, leading to significant deviations between response and correlation functions. Einstein’s relation is no more satisfied at packing fractions greater or equal to 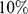. The comparison between panels (b) and (c) of Figure 2, better visible in their insets, shows that the amount of violation increases with the packing fraction.

The GFDT discussed above, Eq. (3), accounts for all the observations of Fig. 2. In our case it reads.
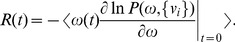
(4)


**Figure 3:**
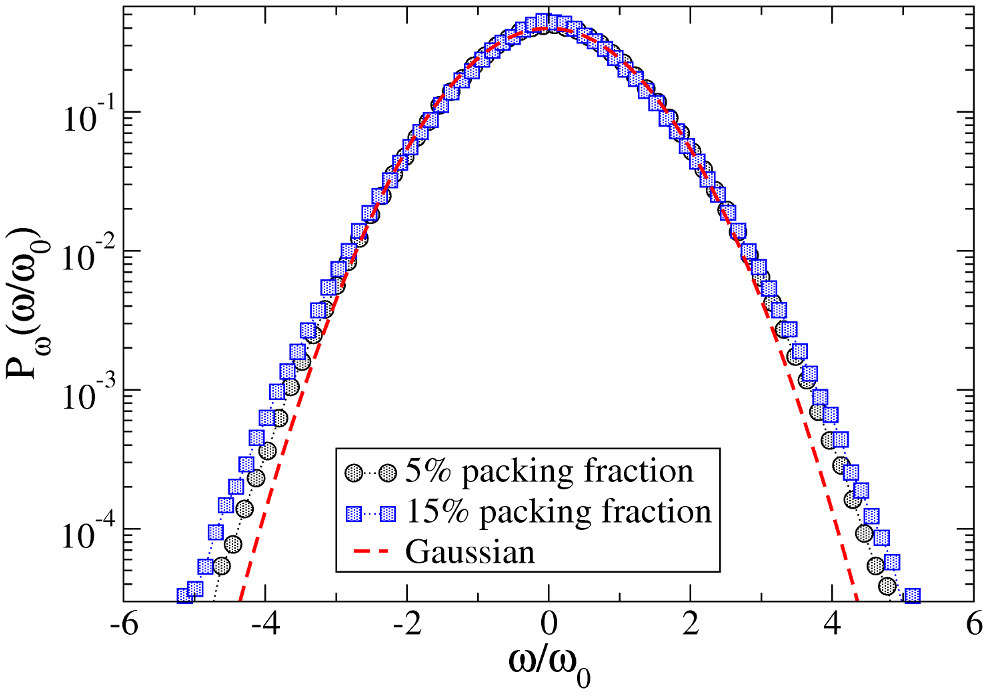
Velocity distributions. PDF of the rotator’s angular velocity rescaled by 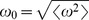 for low (black circles, 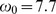 rad/s) and high (blue squares, 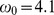 rad/s) densities. The red dashed line shows a Gaussian fit for comparison.



The static properties of the system are fully described by the joint probability density function (PDF) 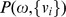 of 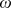 and of the gas particle velocities 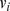, with 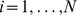. In Figure 3, we show the PDF 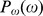 of the angular velocity of the rotator for different gas densities. It corresponds to the *marginalized* 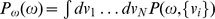 of the joint PDF. The determination of the complete joint PDF is out of the scope of our experimental apparatus. However, steps in this direction are discussed at the end of the paper. Deviations from a Gaussian, in the PDF of the rotator’s angular velocity, appear at all densities. Such discrepancies include a slightly enhanced peak at small velocity, due to the presence of dry friction [24], as well as tails slightly larger than Gaussian at high velocities, whose origin is likely to be the inelasticity of collisions. A good fit of 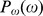 may be obtained in the form of.

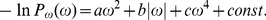
(5)The parameters of the fits in the three cases are 
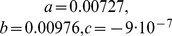
 for 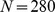; 
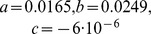
 for 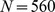; 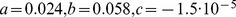 for 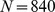. Units for 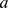, 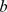 and 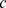 are 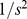, 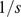 and 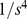 respectively. Negative values for coefficient 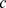 are of course non-physical at very high velocities, however they give reason of a good fit in the observable range; one may imagine that further corrections at higher order (irrelevant in this study) are present.

Assuming a factorization among 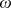 and 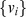, i.e. 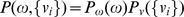, one has.
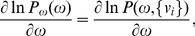
(6)


that used with (5) for the GFDT gives 
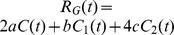
, with 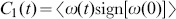 and 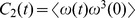. The non-Gaussian form of the PDF clearly modifies the relation between response and correlation. However, as already observed in molecular dynamics simulations [25], it may happen that the “extra” correlators 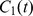 and 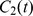 coming from non-Gaussianity do not deviate substantially (once normalized to be 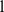 at the origin, 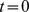) from the velocity-velocity correlation function 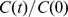. Our experiment shows clearly, see Fig. 2 (in particular the curves with green diamonds), that in all cases (dilute and more dense) the correction induced only by non-Gaussian terms is very small, i.e. 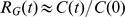. The first implication of this is that our experiment is in agreement with the GFDT in the dilute case (Fig. 2a). The second implication is that the breakdown of Einstein’s relation can only be imputed to the failure of assumption (6) in the more dense cases (Fig. 2b and 2c).

### Coupling with the Fluid

**Figure 4:**
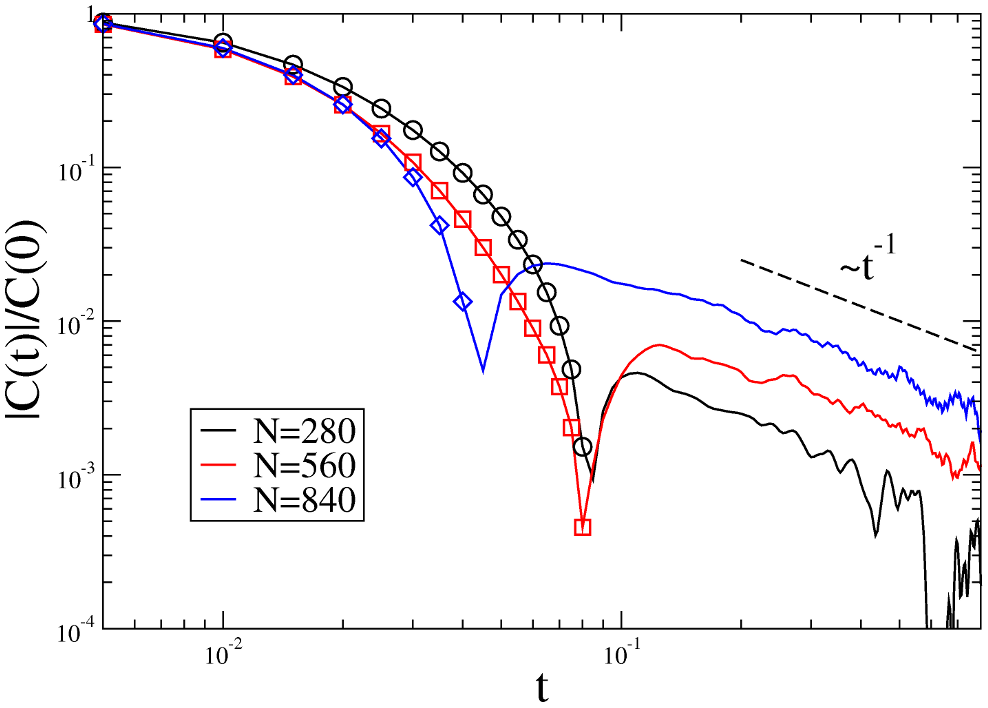
Long tails. Absolute value of autocorrelations in log-log scale (symbols denote positive values) for different densities.

The emergence of the relevance of coupling between wheel and fluid, going from the dilute case to the dense one, already appears in the study of autocorrelations functions. At low packing fraction, the shape of 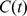 is dominated by a single exponential decay with an almost negligible negative part which displays a power law decay at large times. The presence of a time interval with 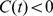 and the final power law decay become more and more important as the density is increased. In Figure 4, we plot 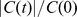 in log-log scale for different densities. At each density a time 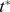 exists where 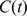 change sign, from 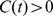 to 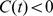, well evident in Fig. 4 as a sudden change of the derivative. The negative region is reminiscent of backscattering phenomenon and characterizes also equilibrium molecular fluids with memory effects arising at high density. The slow final decay 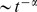 with 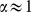 is analogous to the phenomenon of long-time tails whose existence is acknowledged in granular systems [26] and is due to the coupling of the tracer’s density with the fluid’s shear flow [27]. Both the negative region and the power-law decay become more and more relevant as the density is increased.

Both these features imply the existence of more than one time-scale. In a molecular fluid at equilibrium, however, even when 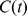 shows such a non-trivial behavior, particles velocities remain statistically independent as a consequence of 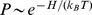, so that Eq. (6) holds and Einstein’s relation remains satisfied. Out of equilibrium, on the other side, the coupling between rotator and particles, suggested by the multiscale behavior, induce velocity correlations among different degrees of freedom [28]. Such an entangled joint PDF can no more be replaced by the marginalized 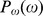, in Eq. (4): its ultimate consequence is the breakdown of Einstein’s relation. In our experiment the presence of slowly-decaying correlations is present at all values of the density. However, we point out that such correlations intensify with the increase of density. As a consequence, it is plausible that the observed violation of Einstein's relation is due to the appearance of internal correlations that becomes important when the density is increased. This hypotesis is also supported by the study, discussed in the following, of rotator-gas correlations.

**Figure 5:**
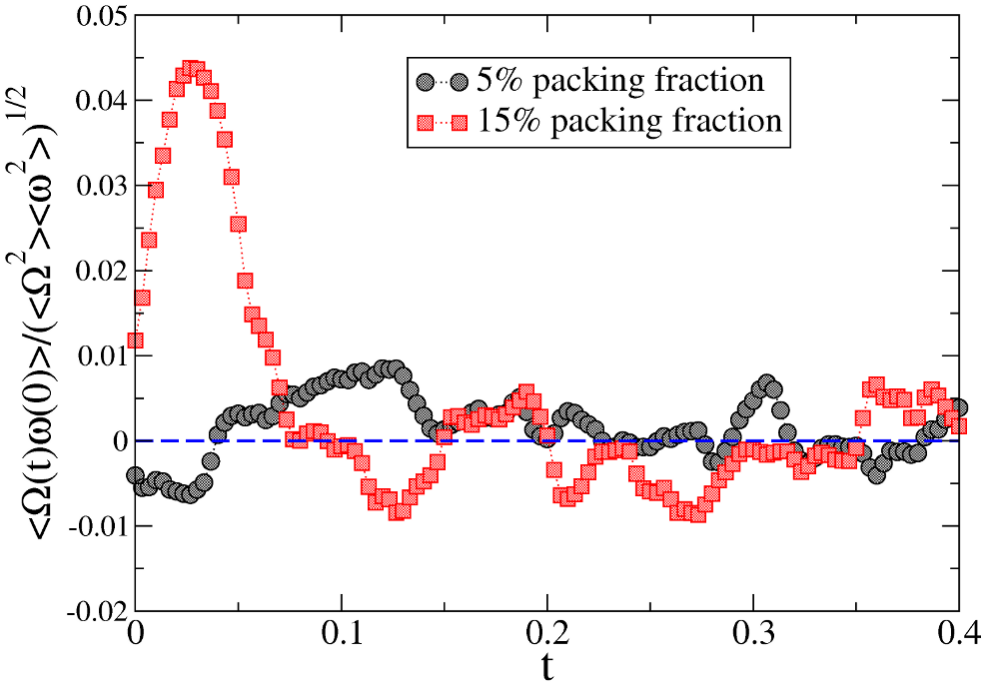
Coupling with the gas. Correlation between the angular velocity of the probe 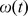 and the average angular velocity of the fluid 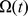 (see text for definition) for the most dilute and the most dense experiments.

In order to find an explicit form for the correlation functions appearing in the GFDT, Eq. (4), it is necessary to understand the role of the relevant degrees of freedom coupled with 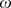. In certain cases, it has been shown that the dominant contribution of this coupling consists in a “hydrodynamic” velocity field (related to gas particles surrounding the wheel) [25], [28], [29]. The correlation between the rotator and such a local velocity field implies a correction to Eq. (6) and, therefore, to Einstein's relation; the physical meaning is the emergence in the dynamics of the rotator of another timescale related to the typical relaxation time of the local field fluctuations. In Fig. 5, we have verified the existence in the dense regime of such a coupling by plotting the cross-correlation 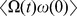, where 
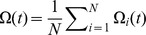
 and 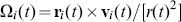 is the angular velocity of particle 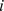 at position 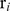 relative to the center of rotation. The same measurement (properly rescaled) reveals a much less evident coupling in the dilute configuration. This is a strong evidence that correlations between 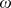 and 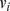 are relevant and, therefore, Eq. (6) does not hold. The fair coincidence in time of the maximum of the cross-correlation with the region of maximum violation of Einstein’s relation (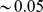 seconds) corroborates our argument. The attempt to fit responses and correlations through a simple model [28] with two linearly coupled stochastic variables (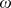 and 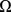) had negative results: the behavior of our experiment is rather complex and it is difficult even providing a conjecture for the functional shape of 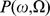. Indeed, the slow decay of autocorrelations at large times is a phenomenon which is incompatible with a simple linear model. There is the need of a more refined kinetic theory, possibly in terms of perturbative expansions, such as the Mode Coupling Theory [30], and tailored to our two-component system (wheel and granular gas) characterised by two different, yet coupled, kinetic temperatures.

## Methods

The granular medium, made of 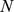 non-magnetic steel beads, diameter 4 mm and mass 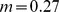 g, is housed in a polymethyl-methacrylate (PMMA) cylinder (diameter 90 mm) with a conical-shaped floor. A fixed holder encloses a miniaturized angular encoder (model AEDA-3300 by Avago Technologies). The encoder, which also supports the rotator (see below), provides high resolution measurements (up to 80,000 division/revolution at the maximum rate of 20 kHz) of the rotator position. The encoder is used at one half of its maximum sensitivity that corresponds to a resolution of 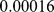 rads, with an acquisition rate of 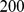 samples per second. The cylinder is vibrated by an electrodynamic shaker (model V450 by LDS Test & Measurement) fed by a sinusoidal excitation. An accelerometer measures the actual acceleration induced to the system. A high-speed camera (EoSens CL by Mikrotron) tracks single beads at 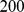 frames per second, in order to measure their velocity: uncertainty in the determination of the centre of mass of spheres is estimated to be 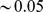 mm [22]. A PMMA rectangular parallelepiped, termed “wheel” in the paper, of height 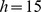 mm and rectangular base with dimensions 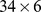 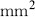 is suspended, by a rod through a small hole in the top face, to the angular encoder that records the wheel’s angle. The momentum of inertia of the free rotator (cylinder plus rod) is 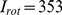 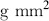. The setup is similar to that used in Ref. [22] with the addition of a miniaturized dc motor (model 108–105 from Precision Microdrives) connected, through a couple of gears, to the rotation axis of the wheel. We have not measured the total momentum of inertia 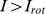 of the rotator coupled to the motor. The motor is driven by sharp rectangular electrical pulses provided by the acquisition board (model NI USB-6353 from National Instruments) through a simple voltage buffer circuit. The effect of the pulses is to perturb the rotator’s velocity that is the variable taken in consideration here. We use 2 ms long and 5 V high pulses provided to the motor every second. We have verified that both the response and correlation functions take less than one second to go to zero, i.e. all perturbations can be considered independent. We have also directly checked the linear response regime. In order to have clean response measurements, we performed 70 hours long experiments. The acquisition rate of the system is set at 200 Hz. We use three different gas densities (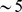
%, 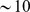
% and 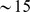
% of the total volume) varying the number of beads (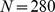, 560 and 840, respectively). By a careful particle tracking procedure [22] we can measure one of the horizontal components (on the plane) of the particles’ velocity 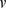, which gives access to the so-called granular temperature 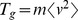. Our choice to employ a wheel which is free to rotate around a fixed (vertical) axis, instead of a torsion oscillator [21], is only motivated by simplicity of realization. Of course, such different choice is irrelevant for the regime of linear response.

At the currently used maximum acceleration (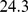 in units of gravity acceleration), the typical horizontal velocity 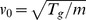 of particles goes from 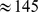 mm/s at the maximum density (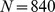) to 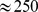 mm/s at the minimum one (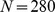). Estimates of the particle-particle mean free path give 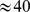 mm for the more dilute experiment and 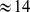 mm for the more dense one. The estimate for the mean free time for particle-particle collisions goes from 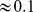 to 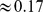 seconds, for the more dense and the more dilute experiment respectively. The mean free time of the rotator (which does not distinguish between different particles) is 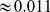 seconds in the most dilute experiment and 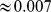 seconds in the most dense one.
